# Longitudinal patterns and predictors of healthcare utilization among cancer patients on home-based palliative care in Singapore: a group-based multi-trajectory analysis

**DOI:** 10.1186/s12916-022-02513-y

**Published:** 2022-09-22

**Authors:** Qingyuan Zhuang, Poh-Heng Chong, Whee Sze Ong, Zhi Zheng Yeo, Cherylyn Qun Zhen Foo, Su Yan Yap, Guozhang Lee, Grace Meijuan Yang, Sungwon Yoon

**Affiliations:** 1 Division of Supportive and Palliative Care, National Cancer Centre Singapore, 11 Hospital Drive, Singapore, 169610 Singapore; 2 HCA Hospice, Singapore, Singapore; 3 Division of Clinical Trials and Epidemiological Sciences, National Cancer Centre Singapore, Singapore, Singapore; 4 Palliative Care Services, Department of Geriatric Medicine, Changi General Hospital, Singapore, Singapore; 5 Department of Internal Medicine, Singapore General Hospital, Singapore, Singapore; 6 Lien Centre for Palliative Care, Duke-NUS Medical School, Singapore, Singapore; 7 Health Services and Systems Research, Duke-NUS Medical School, Singapore, Singapore; 8 Centre for Population Health Research and Implementation, Singapore Regional Health System, Singapore, Singapore

**Keywords:** Oncology, Home-based palliative care, End-of-life, Trajectory analyses, Healthcare utilization

## Abstract

**Background:**

Home-based palliative care (HPC) is considered to moderate the problem of rising healthcare utilization of cancer patients at end-of-life. Reports however suggest a proportion of HPC patients continue to experience high care intensity. Little is known about differential trajectories of healthcare utilization in patients on HPC. Thus, we aimed to uncover the heterogeneity of healthcare utilization trajectories in HPC patients and identify predictors of each utilization pattern.

**Methods:**

This is a cohort study of adult cancer patients referred by Singapore Health Services to HCA Hospice Service who died between 1st January 2018 and 31st March 2020. We used patient-level data to capture predisposing, enabling, and need factors for healthcare utilization. Group-based multi-trajectory modelling was applied to identify trajectories for healthcare utilization based on the composite outcome of emergency department (ED) visits, hospitalization, and outpatient visits.

**Results:**

A total of 1572 cancer patients received HPC (median age, 71 years; interquartile range, 62–80 years; 51.1% female). We found three distinct trajectory groups: group 1 (31.9% of cohort) with persistently low frequencies of healthcare utilization, group 2 (44.1%) with persistently high frequencies, and group 3 (24.0%) that begin with moderate frequencies, which dropped over the next 9 months before increasing in the last 3 months. Predisposing (age, advance care plan completion, and care preferences), enabling (no medical subsidy, primary decision maker), and need factors (cancer type, comorbidity burden and performance status) were significantly associated with group membership. High symptom needs increased ED visits and hospitalizations in all three groups (ED visits, group 1–3: incidence rate ratio [IRR] 1.74–6.85; hospitalizations, group 1–3: IRR 1.69–6.60). High home visit intensity reduced outpatient visits in all three groups (group 1–3 IRR 0.54–0.84), while it contributed to reduction of ED visits (IRR 0.40; 95% CI 0.25–0.62) and hospitalizations (IRR 0.37; 95% CI 0.24–0.58) in group 2.

**Conclusions:**

This study on HPC patients highlights three healthcare utilization trajectories with implications for targeted interventions. Future efforts could include improving advance care plan completion, supporting care preferences in the community, proactive interventions among symptomatic high-risk patients, and stratification of home visit intensity.

**Supplementary Information:**

The online version contains supplementary material available at 10.1186/s12916-022-02513-y.

## Background

Healthcare utilization in patients with cancer escalates at the end of life. This is primarily driven by hospital visits that drive up healthcare-related costs [1–6]. Beyond economic repercussions, the experience of repeated hospital visits at EOL can result in care fragmentation and non-beneficial interventions [7–9]. There is compelling evidence that providing home-based palliative care (HPC) services can moderate the problem of rising healthcare utilization by reducing symptom burden, decreasing the number and length of hospital visits and increasing the chances of dying at home [10, 11]. Broadly, HPC services offer holistic care to patients at home or community-based care facilities through in-person visits up to several times a week, provision of supplies and equipment, and round-the-clock telephone support. Specific referral criteria may differ across services and countries, but referred patients are generally stable, with poor performance status and limited lifespan [12, 13]. Despite HPC support, research on HPC enrolees suggests that a proportion continues to have repeated ED visits and hospitalizations [14, 15]. To optimize HPC outcomes through reducing unnecessary hospital visits, it is imperative to first understand the heterogeneity in healthcare utilization among HPC patients and the reasons driving the differences.

Within literature, predictive factors for higher utilization among HPC patients included younger age [14–16], male gender [14, 15, 17], more comorbidities [14, 16], better function [17], and reduced hospice capacity [14, 15, 18]. Preference to die at home, having an involved decision-maker, and an advanced care plan (ACP) protect against hospitalizations [17, 19, 20]. These studies typically measured healthcare utilization as an aggregated single composite index over a cross-sectional period (e.g. ≥ 2 hospitalizations in the last month of life). Such approaches oversimplify the complex intra- and inter-individual variability of the real-life clinical context [8, 21]. To our knowledge, differential patterns of healthcare utilization over the HPC journey and the factors influencing the differences have not been elucidated.

Considering the limitations in literature, we adopted group-based multi-trajectory modelling (GBMTM) to uncover groups of HPC patients with different healthcare utilization over time. GBMTM is a specialized finite mixture modelling that allows groups of distinct trajectories to emerge from data as opposed to a conventional method that estimates patient-level averages [22, 23]. Moreover, GBMTM has an advantage of adding time-varying covariates to estimate whether events that occur during the course of a trajectory could alter the trajectory shape itself [24]. Identifying and characterizing groups of HPC patients with meaningfully different healthcare use patterns over time presents unique opportunities for targeted interventions designed to address their varying needs [25].

Therefore, this study aimed to uncover groups with distinct patterns of healthcare utilization, identify factors associated with group membership, and examine the influence of symptom burden and home visit frequency on healthcare utilization within each group.

## Methods

### Setting

The current study was conducted in Singapore where the EOL population segment (80% with cancer) contributes to approximately 50% of healthcare cost per capita of $60,000 (US$44,200) [26]. Ranked 12th in the 2015 Economist Intelligence Unit’s Quality of Death Index, Singapore’s palliative care services are conceptually and operationally similar to many first-world countries elsewhere [27]. Specifically, our HPC model offers patients with a life expectancy of less than 12 months to receive concurrent inter-disciplinary palliative care at home and disease-modifying therapies at tertiary hospitals [28, 29]. This model bears similarities to the Medicare Care Choices Model in the United States and some Hospice at Home services in England [13, 30, 31].

Our HPC services are run by voluntary welfare organizations, funded by government subsidies and philanthropic donations. Most offer care packages comprising home visits, equipment loans and round-the-clock support as core elements. Around 60% of Singapore’s HPC caseload is managed by HCA Hospice (HCA). HCA serves more than 3000 patients a year, of which 80% have cancer diagnoses. The free service includes an after-hours helpline for medical crisis. Enrolled patients are managed by multidisciplinary teams comprising of palliative care doctors, nurses, medical social workers, counsellors, and volunteers. Bereavement support is also provided in selected cases [32].

### Study design and participants

This is a cohort study of cancer patients in HCA who died between 1st January 2018 and 31st March 2020. We included Singapore citizens or permanent residents referred to HCA by Singapore Health Services Regional Health System (SingHealth RHS). Out of the three healthcare clusters, SingHealth RHS is the largest in Singapore serving 70% of public sector cancer patients within inpatient and outpatient settings.

Electronic health records (EHR) from both HCA [32] and SingHealth [33] databases were extracted and linked using the patient’s unique National Registration Identity Card number. Linked datasets were de-identified before statistical analysis.

### Outcome measures and covariates

Outcome sought a priori was healthcare utilization trajectories 1 year after HPC enrolment. We assumed stratification of trajectories to split along differential patterns of health service use. Emergency department (ED) visits, hospitalizations, and outpatient visits were chosen as the three healthcare utilization indicators of interest as they have been shown to rise substantially at EOL among cancer patients [34].

We identified covariates for testing association with outcomes by referencing Andersen’s model for health service use [35] (Additional file 1: Table S1). Andersen’s landmark model suggests health service use as a function of (1) predisposition to use services, (2) enabling or impeding factors, and (3) need for care. This model is widely used to investigate factors associated with healthcare utilization [36, 37].

Predisposing factors included age, gender, ethnicity, religion, marital status, residential status, and health beliefs. Andersen defines health beliefs as attitudes, values, and knowledge a patient has about health and health services [35]. For this, we included the patient’s awareness of diagnosis and prognosis, family’s awareness of diagnosis and prognosis, and ACP components. ACP components included the preferred place of care, preferred place of death and preferred plan of care (Additional file 1: Tables S1 and S2).

Enabling or impeding factors included socioeconomic (SES) surrogates of the Housing value Index (HI) and medical subsidy testing categories. HI utilizes housing type as a surrogate of past SES, where eligibility for public housing type is based on income ceilings [38–40] (Additional file 1: Table S3). Medical subsidy testing calculates government healthcare subsidies, taking into consideration current per capita household income [41]. Subsidies were categorized into 0% (highest SES), 1–25%, 25–50%, and 51–80% (lowest SES). Additional factors included social structure variables pertaining to primary caregiver, primary decision maker, and prevailing living arrangements.

Need-related factors included cancer type, comorbid burden, and Eastern Cooperative Oncology Group (ECOG) performance status. Cancer types were grouped by two-digit International Classification of Diseases, Tenth Revision, Clinical Modification (ICD-10-CM) codes [42]. Comorbidity burden was computed using Charlson Comorbidity Index (CCI) [43]. Additional factors included time-varying symptoms and psychosocial needs. HCA assesses the patient’s symptoms and/or psychosocial severity at every home visit. Details on symptoms and psychosocial assessment are in Additional file 1: Table S4a and S4b. We predefined high symptom needs by an assessment of “U1” and high psychosocial needs as an assessment of “P3” or “P4”. We measured home visit intensity using visit counts, with visits after-hours weighted twice as much, i.e. two points assigned. We predefined high intensity as ≥ 4 points over 2 weeks, as minimal standards are pegged at one home visit within 1 to 2 weeks.

STROBE guidelines were referenced in reporting this study [44].

### Statistical analysis

First, we determined trajectories for the number of ED visits, hospitalizations, and outpatient visits separately using group-based trajectory modelling (GBTM), with extensions to account for non-random patient attrition due to death [22, 45]. GBTM uses maximum likelihood to identify latent subgroups of individuals with similar trajectories for a variable and has been used to describe healthcare trajectories in other populations [46–48]. Details of the trajectory analyses are in the additional file. Briefly, we used Poisson distribution to model each measure with time expressed in terms of weekly, bi-weekly, thrice-weekly, and monthly. For each time-unit analysis, we excluded patients who died within the first time-unit interval. The best-fit model for each time-unit was selected based on the Bayesian Information Criterion (BIC). To determine the optimum model for each measure, we compared cohort size and BIC between the best-fit model of the 4 time-units and identified the time-unit that provided a balance between information loss and goodness of model fit.

Second, we applied group-based multi-trajectory modelling (GBMTM) to identify distinct trajectories for healthcare utilization based on the composite outcome of ED, hospitalization, and outpatient visits [23]. GBMTM is a generalization of GBTM allowing multiple variables measuring an outcome to be jointly analysed. The optimum GBMTM model is again determined based on the BIC. We classified patients into one of the trajectory groups of the optimum GBMTM model based on maximum posterior probability assignment rule. We assessed model performance using these criteria: (1) close correspondence between the model’s estimated group size and the actual percentage of patients classified into each group, (2) high (>0.7) average posterior probabilities of group membership, (3) sufficient patients (>5% in proportion) classified in each group, and (4) narrow confidence band for each group [24].

Third, we identified predictors for trajectory group membership in the optimum GBMTM model using multinomial logit regression. We also assessed whether time-varying symptom burden, psychosocial needs and home visit intensity were associated with a within-group change in healthcare utilization trajectory. These associations were tested by expanding the Poisson regression model specification of each group-specific trajectory to include indicator variables measuring each patient’s presence of high symptom needs, high psychosocial needs, and high intensity of home visits at each time-unit of analysis. For these analyses, all statistically significant covariates on univariate analysis were considered for inclusion in the multivariable model. To avoid model overfitting and multi-collinearity, pairwise Spearman’s correlations (*ρ*) between covariates were generated and clinical judgement was used to decide which highly correlated covariates (*ρ* >0.65) to drop from the final multivariable model.

There was a low percentage of patients with missing data for the covariates. Patients with missing values for a covariate were retained in the multinomial logit regression analyses and analysed as a category for that covariate in these analyses. No imputation was performed. The total number of predictors tested was below the maximum number of predictors that could be fitted given the total sample size and number of patients in each trajectory group [49]. Analyses were performed using SAS 9.4 (SAS Institute Inc., Cary, NC), with GBTM and GBMTM conducted using PROC TRAJ macros. All statistical tests were 2-sided with a 5% significance level.

## Results

### Group-based trajectory analysis of hospitalization, ED, and outpatient visits separately

**Fig. 1:**
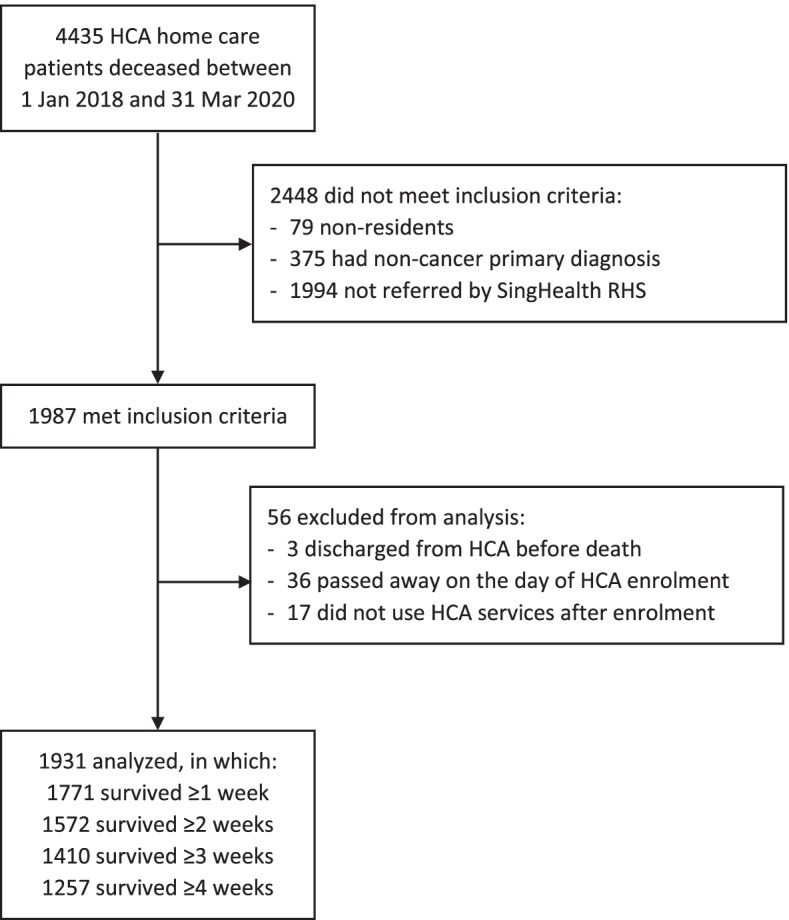
Study participant flowchart

A total of 1931 patients were in the analysis cohort (Fig. 1). Of these patients, 1771, 1572, 1410, and 1257 were included based on weekly, bi-weekly, thrice-weekly, and monthly time-unit intervals, respectively (Additional file 1: Figs. S1–S3) The optimum model of each outcome was comprised of three groups with bi-weekly time-unit intervals.

### Group-based multi-trajectory analysis of healthcare utilization as a composite measure

**Fig. 2:**
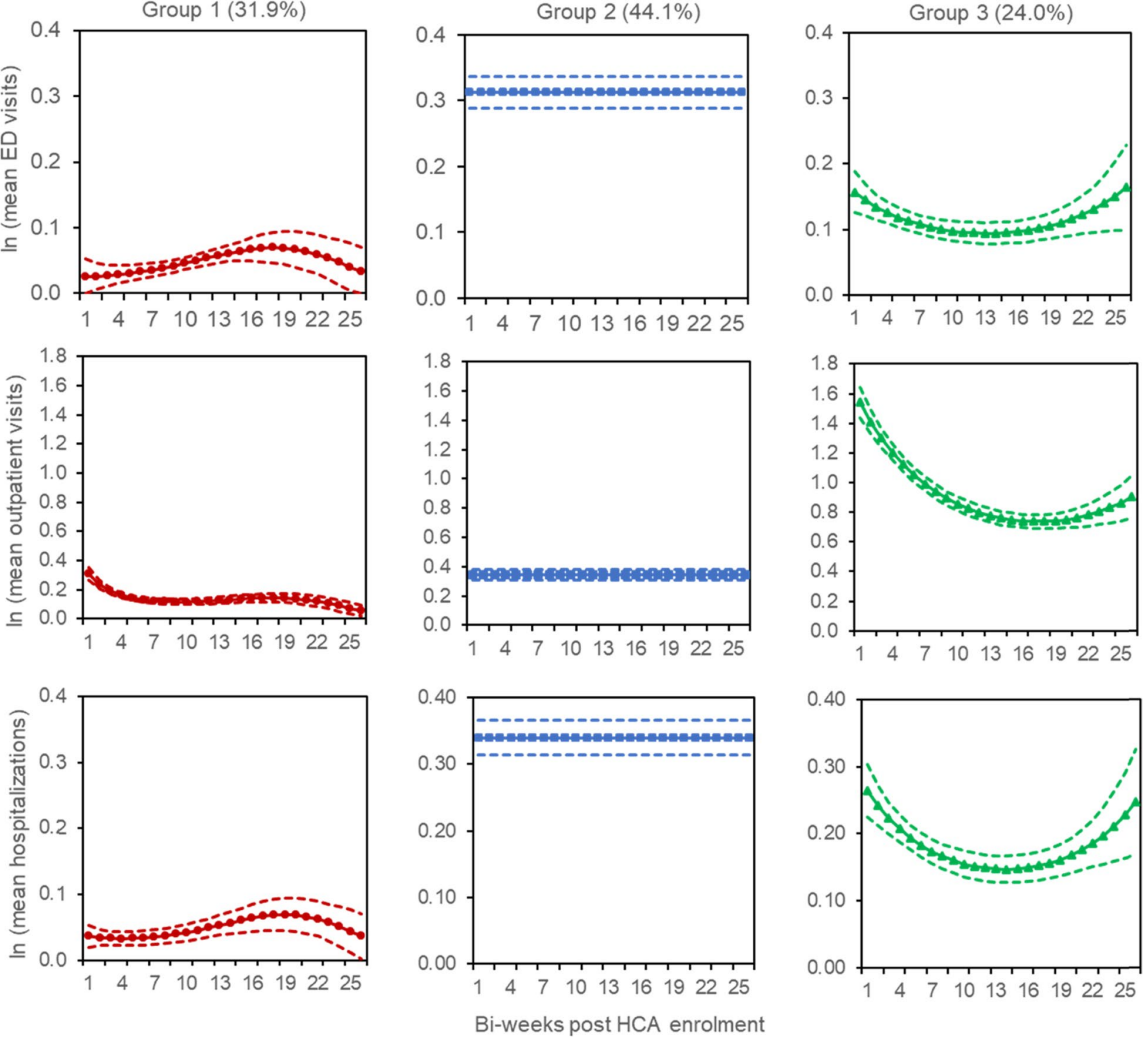
Trajectories of healthcare utilization (emergency department visits, outpatient visits, and hospitalizations) using group-based multi-trajectory modelling

We included 1572 patients in the GBMTM analysis (Table 1). Figure 2 illustrates the optimum model for our composite measure. Group 1 (31.9% of patients) was characterized by low frequencies of ED visits, outpatient visits, and hospitalizations. Trajectory shape of ED visits and hospitalizations increased slightly over the first 9 months before decreasing in the last 3 months, while the outpatient visits saw a steady decline over the 12-month period. Group 2 (44.1%) was characterized by high frequencies of ED visits and hospitalizations, and moderate frequencies for outpatient visits, which remained constant over time. Group 3 (24.0%) was characterized by moderate frequencies of ED visits and hospitalizations and high frequencies of outpatient visits. The trajectory shape of each visitation type dipped over the first 9 months before increasing in the last 3 months.

**Table 1:** Baseline characteristics separated by trajectory groups^a^

Variable	Group 1		Group 2		Group 3		Total	
	No.	%	No.	%	No.	%	No.	%
Overall	551	100.0	664	100.0	357	100.0	1572	100.0
Age, years^b^	74 (65–83)		70 (63–79)		66 (58–75)		71 (62–80)	
Gender								
Male	267	48.5	340	51.2	162	45.4	769	48.9
Female	284	51.5	324	48.8	195	54.6	803	51.1
Ethnicity								
Chinese	444	80.6	525	79.1	282	79.0	1251	79.6
Malay	59	10.7	71	10.7	38	10.6	168	10.7
Indian	27	4.9	40	6.0	20	5.6	87	5.5
Others	21	3.8	28	4.2	17	4.8	66	4.2
Religion								
Christianity (all denominations)	107	19.4	126	19.0	76	21.3	309	19.7
Buddhism	247	44.8	305	45.9	154	43.1	706	44.9
Taoism	50	9.1	50	7.5	35	9.8	135	8.6
Islam	87	15.8	103	15.5	51	14.3	241	15.3
Hinduism	15	2.7	25	3.8	13	3.6	53	3.4
Others	11	2.0	16	2.4	8	2.2	35	2.2
No religion	31	5.6	37	5.6	19	5.3	87	5.5
Missing	3	0.5	2	0.3	1	0.3	6	0.4
Marital status								
Single	27	4.9	51	7.7	25	7.0	103	6.6
Married	345	62.6	455	68.5	258	72.3	1058	67.3
Separated/divorced	23	4.2	30	4.5	24	6.7	77	4.9
Widowed	156	28.3	128	19.3	50	14.0	334	21.2
Residential status								
Singapore citizen	530	96.2	637	95.9	340	95.2	1507	95.9
Permanent resident	21	3.8	27	4.1	17	4.8	65	4.1
At least one ACP component completed^d^								
No	207	37.6	341	51.4	181	50.7	729	46.4
Yes	344	62.4	323	48.6	176	49.3	843	53.6
Preferred place of care								
Hospital	30	5.4	60	9.0	35	9.8	125	8.0
Home	276	50.1	217	32.7	114	31.9	607	38.6
Others	24	4.4	22	3.3	9	2.5	55	3.5
No preference	9	1.6	17	2.6	7	2.0	33	2.1
Missing	5	0.9	7	1.1	11	3.1	23	1.5
ACP not done	207	37.6	341	51.4	181	50.7	729	46.4
Preferred place of death								
Home	270	49.0	194	29.2	108	30.3	572	36.4
Hospice	21	3.8	32	4.8	17	4.8	70	4.5
Others	11	2.0	11	1.7	10	2.8	32	2.0
No preference	37	6.7	69	10.4	33	9.2	139	8.8
Missing	5	0.9	17	2.6	8	2.2	30	1.9
ACP not done	207	37.6	341	51.4	181	50.7	729	46.4
Preferred plan of care								
Full active	1	0.2	8	1.2	5	1.4	14	0.9
Limited intervention	93	16.9	120	18.1	60	16.8	273	17.4
Comfort only	229	41.6	163	24.5	84	23.5	476	30.3
Missing	21	3.8	32	4.8	27	7.6	80	5.1
ACP not done	207	37.6	341	51.4	181	50.7	729	46.4
Patient awareness of diagnosis								
No	226	41.0	227	34.2	89	24.9	542	34.5
Yes	325	59.0	436	65.7	268	75.1	1029	65.5
Missing	0	0.0	1	0.2	0	0.0	1	0.1
Patient awareness of prognosis								
No	304	55.2	319	48.0	149	41.7	772	49.1
Yes	247	44.8	344	51.8	208	58.3	799	50.8
Missing	0	0.0	1	0.2	0	0.0	1	0.1
Family awareness of diagnosis								
No	13	2.4	38	5.7	23	6.4	74	4.7
Yes	538	97.6	625	94.1	334	93.6	1497	95.2
Missing	0	0.0	1	0.2	0	0.0	1	0.1
Family awareness of prognosis								
No	52	9.4	78	11.7	53	14.8	183	11.6
Yes	499	90.6	585	88.1	304	85.2	1388	88.3
Missing	0	0.0	1	0.2	0	0.0	1	0.1
Housing value index^c^								
High	127	23.0	130	19.6	75	21.0	332	21.1
Medium	373	67.7	479	72.1	256	71.7	1108	70.5
Low^c^	51	9.3	55	8.3	26	7.3	132	8.4
Medical subsidy means testing								
0%	110	20.0	153	23.0	96	26.9	359	22.8
1–25%	4	0.7	4	0.6	4	1.1	12	0.8
26–50%	98	17.8	102	15.4	66	18.5	266	16.9
51–80%	320	58.1	386	58.1	182	51.0	888	56.5
Not done	19	3.4	19	2.9	9	2.5	47	3.0
Main caregiver								
Self	4	0.7	7	1.1	12	3.4	23	1.5
Spouse	102	18.5	142	21.4	104	29.1	348	22.1
Relatives (children included in category)	435	78.9	498	75.0	236	66.1	1169	74.4
Others	6	1.1	14	2.1	3	0.8	23	1.5
Missing	4	0.7	3	0.5	2	0.6	9	0.6
Primary decision maker								
Self	4	0.7	7	1.1	12	3.4	23	1.5
Spouse	102	18.5	142	21.4	104	29.1	348	22.1
Children	394	71.5	433	65.2	208	58.3	1035	65.8
Other relative	41	7.4	68	10.2	30	8.4	139	8.8
Others	4	0.7	11	1.7	2	0.6	17	1.1
Missing	6	1.1	3	0.5	1	0.3	10	0.6
Living arrangement								
Alone	17	3.1	26	3.9	19	5.3	62	3.9
With spouse only	56	10.2	89	13.4	46	12.9	191	12.2
With children only	153	27.8	140	21.1	64	17.9	357	22.7
With spouse and children	125	22.7	186	28.0	115	32.2	426	27.1
With relatives	22	4.0	37	5.6	15	4.2	74	4.7
Others	174	31.6	179	27.0	96	26.9	449	28.6
Missing	4	0.7	7	1.1	2	0.6	13	0.8
Primary cancer diagnosis								
Lip, oral cavity, and pharynx	22	4.0	18	2.7	14	3.9	54	3.4
Digestive organs	230	41.7	322	48.5	108	30.3	660	42.0
Respiratory and intrathoracic organs	107	19.4	129	19.4	70	19.6	306	19.5
Breast	36	6.5	40	6.0	39	10.9	115	7.3
Female genital organs	48	8.7	63	9.5	44	12.3	155	9.9
Male genital organs	20	3.6	24	3.6	15	4.2	59	3.8
Urinary tract	25	4.5	18	2.7	18	5.0	61	3.9
Lymphoid, haematopoietic, and related tissue	17	3.1	11	1.7	22	6.2	50	3.2
Others	46	8.3	39	5.9	27	7.6	112	7.1
Charlson Comorbidity Index^b^	8 (7–9)		8 (8–10)		8 (8–9)		8 (8–9)	
ECOG performance status								
0–2	174	31.6	257	38.7	186	52.1	617	39.2
3–4	327	59.3	320	48.2	121	33.9	768	48.9
Not assessed	50	9.1	87	13.1	50	14.0	187	11.9
Survival post enrolment in home hospice care, weeks	10.1 (4.5–21.7)		6.7 (3.8–11.6)		17.5 (8.8–32.6)		9.4 (4.7–19.3)	

*Abbreviation*: *ACP* advanced care planning, *ECOG* Eastern Cooperative Oncology Group

^a^Group 1 = persistently low emergency visits, outpatient visits and hospitalizations, group 2 = persistently high emergency visits and hospitalizations with moderately high outpatient visits, group 3 = high outpatient visits and moderately high emergency visits and hospitalizations which all dip before rising over time

^b^Data presented is median (interquartile range)

^c^Housing value index low corresponds to high housing subsidy (public HDB 1–2 rooms flat), medium to moderate housing subsidy (public HDB 3–4 rooms flat), and high to minimal or no housing subsidy (public HDB 5-room or larger flats and private housing)

^d^ACP components are (1) preferred place of care, (2) preferred place of death, and (3) preferred plan of care

The performance of the optimum model was good, with average posterior probabilities of patients classified in each group greater than 0.8 (Additional file 1: Table S5). Table 1 provides patient characteristics breakdown by trajectory group.

### Factors associated with healthcare utilization trajectories

We used group 1 as the reference group in the multinomial logit model. Patients who were younger (relative risk ratio [RRR] 1.02; 95% CI 1.01–1.04 per year decrease), had higher CCI (RRR 1.16; 95% CI 1.10–1.21 per score increase), had no ACP (RRR 3.35; 95% CI 2.29–4.91), or had preferred care plans for a full treatment or limited interventions (RRR 2.32; 95% CI 1.45–3.69) were at increased risk of group 2 membership. Patients with breast (RRR 0.35; 95% CI 0.18–0.70) or urinary tract cancers (RRR 0.39; 95% CI 0.17–0.90) or received 1–50% medical subsidy (RRR 0.65; 95% CI 0.43–0.97) were less likely to be in group 2 (Table 2).

**Table 2:** Predictors of group membership

Variable	Relative risk ratio (95% CI)			
	Base category for multinomial logit model = group 1			
	Univariable analysis		Multivariable analysis^1^	
	Group 2	Group 3	Group 2	Group 3
Age (per year decrease)	**1.03 (1.02–1.04)**	**1.07 (1.05––1.08)**	**1.02 (1.01–1.04)**	**1.05 (1.04–1.06)**
Gender (ref: male )				
Female	0.80 (0.60–1.06)	1.05 (0.78–1.43)		
Ethnicity (ref: Chinese)				
Malay	1.02 (0.64–1.60)	0.87 (0.51–1.46)		
Indian	1.21 (0.64–2.29)	1.18 (0.60–2.33)		
Others	1.46 (0.69–3.06)	1.35 (0.62–2.96)		
Religion (ref: Christianity)				
Buddhism	1.06 (0.72–1.56)	0.91 (0.60–1.38)		
Taoism	0.76 (0.42–1.38)	0.97 (0.54–1.73)		
Islam	1.02 (0.63–1.63)	0.73 (0.43–1.24)		
Others (including Hinduism)	1.32 (0.64–2.70)	1.21 (0.57–2.57)		
No religion	0.98 (0.51–1.88)	0.89 (0.42–1.87)		
Missing	Note^2^			
Marital status (ref: married)				
Single	1.27 (0.69–2.34)	1.43 (0.75–2.70)	1.25 (0.57–2.73)	0.93 (0.42–2.05)
Separated/divorced	0.95 (0.45–2.00)	1.58 (0.78–3.20)	0.78 (0.31–2.00)	1.05 (0.47–2.35)
Widowed	**0.54 (0.39–0.75)**	**0.35 (0.24–0.53)**	0.90(0.57–1.43)	0.73 (0.45–1.19)
Residential status (ref: citizen)				
Permanent resident	1.26 (0.58–2.71)	1.6 (0.75–3.46)		
At least one ACP component completed* (ref: yes)				
No	**2.20 (1.64–2.96)**	**2.00 (1.46–2.75)**	Note^3^	
Preferred place of care (ref: home)				
Hospital	**2.72 (1.53–4.83)**	**3.31 (1.82–6.02)**	Note^3^	
Others (including No preference)	1.32 (0.72–2.42)	1.07 (0.53–2.17)		
ACP not done	**2.67 (1.94–3.67)**	**2.54 (1.80–3.59)**		
Missing	Note^2^			
Preferred place of death (ref: home)				
Hospice	**2.02 (1.04–3.91)**	1.95 (0.93–4.07)	Note^3^	
Others (including No preference)	**2.39 (1.46–3.91)**	**2.38 (1.42–3.99)**		
ACP not done	**2.98 (2.15–4.13)**	**2.62 (1.85–3.70)**		
Missing	Note^2^			
Preferred plan of care (ref: comfort only)				
Fully active/limited intervention	**2.04 (1.35–3.07)**	**1.98 (1.26–3.10)**	**2.32 (1.45–3.69)**	**2.08 (1.31–3.30)**
ACP not done	**3.05 (2.17–4.29)**	**2.87 (1.99–4.15)**	**3.35 (2.29–4.91)**	**2.74 (1.91–3.93)**
Missing	**2.45 (1.21–4.94)**	**3.82 (1.90–7.70)**	**3.15 (1.44–6.91)**	**4.05 (1.93–8.50)**
Patient awareness of diagnosis (ref: yes)				
No	**0.74 (0.56–0.99)**	**0.44 (0.33–0.59)**	1.2 (0.69–2.08)	0.75 (0.45–1.24)
Missing	Note^2^		Note^2^	
Patient awareness of prognosis (ref: yes)				
No	**0.67 (0.50–0.89)**	**0.53 (0.39–0.72)**	0.66 (0.38–1.14)	0.90 (0.56–1.43)
Missing	Note^2^		Note^2^	
Family awareness of diagnosis (ref: yes)				
No	**3.21 (1.29–7.99)**	**4.21 (1.68–10.54)**	2.23 (0.73–6.81)	2.02 (0.72–5.68)
Missing	Note^2^		Note^2^	
Family awareness of prognosis (ref: yes)				
No	1.10 (0.69–1.75)	**1.76 (1.12–2.78)**	0.79 (0.41–1.54)	1.03 (0.57–1.87)
Missing	Note^2^		Note^2^	
Housing value index (ref: low)				
Medium	1.16 (0.71–1.89)	1.46 (0.83–2.56)		
High	0.92 (0.53–1.60)	1.19 (0.63–2.23)		
Medical subsidy means testing (ref: 51–80%)				
1–50%	0.93 (0.63–1.38)	1.31 (0.88–1.97)	**0.65 (0.43–0.97)**	0.88 (0.67–1.16)
0%	1.17 (0.81–1.69)	**1.73 (1.19–2.53)**	1.18 (0.79–1.76)	**1.57 (1.08–2.28)**
Not done	0.96 (0.41–2.26)	0.91 (0.36–2.32)	0.82 (0.30–2.24)	0.41 (0.14–1.19)
Main caregiver (ref: sSpouse)				
Relatives (children included in category)	0.72 (0.50–1.04)	**0.48 (0.33–0.69)**	Note^3^	
Others (including self)	1.45 (0.50–4.22)	1.51 (0.52–4.36)		
Missing	Note^2^			
Primary decision maker (ref: spouse)				
Children/relatives	0.72 (0.50–1.05)	**0.48 (0.33–0.70)**	1.35 (0.94–1.94)	1.17 (0.96–1.44)
Others (including self)	1.63 (0.50–5.29)	1.86 (0.58–5.97)	2.76 (0.69–11.05)	**3.78 (1.04–13.73)**
Missing	Note^2^		Note^2^	
Living arrangement (ref: with spouse only)				
With children only	**0.51 (0.31–0.86)**	**0.45 (0.25–0.80)**	0.63 (0.35–1.11)	0.74 (0.46–1.18)
With spouse and children	1.07 (0.62–1.83)	1.31 (0.74–2.33)	0.96 (0.58–1.61)	0.87 (0.61–1.23)
With relatives	0.92 (0.42–2.00)	0.80 (0.33–1.93)	0.58 (0.23–1.45)	0.46 (0.19–1.08)
Others (including alone)	**0.58 (0.35–0.96)**	0.69 (0.40–1.19)	0.71 (0.42–1.19)	**0.62 (0.42–0.92)**
Missing	Note^2^		Note^2^	
Primary cancer diagnosis (ref: digestive organs)				
Lip, oral cavity, and pharynx	0.63 (0.29–1.41)	1.39 (0.61–3.17)	0.72 (0.29–1.75)	1.14 (0.47–2.77)
Respiratory and intrathoracic organs	0.78 (0.53–1.15)	1.42 (0.92–2.19)	0.81 (0.53–1.23)	1.44 (0.94–2.21)
Breast	0.63 (0.34–1.16)	**2.08 (1.16–3.71)**	**0.35 (0.18–0.70)**	1.36 (0.75–2.47)
Female genital organs	0.66 (0.39–1.10)	**1.86 (1.11–3.13)**	0.64 (0.34–1.21)	1.49 (0.82–2.71)
Male genital organs	0.92 (0.44–1.94)	1.58 (0.68–3.65)	0.68 (0.31–1.48)	1.47 (0.65–3.32)
Urinary tract	**0.36 (0.17–0.78)**	1.17 (0.58–2.35)	**0.39 (0.17–0.90)**	1.37 (0.63–2.98)
Lymphoid, haematopoietic, and related tissue	0.45 (0.15–1.34)	**3.12 (1.4–6.95)**	0.66 (0.2–2.18)	**4.86 (2.20–10.74)**
Others	**0.45 (0.25–0.80)**	1.11 (0.62–1.98)	0.62 (0.32–1.19)	1.01 (0.54–1.89)
Charlson Comorbidity Index (per score increase)	**1.20 (1.13–1.27)**	1.03 (0.97–1.08)	**1.16 (1.10–1.21)**	**1.03 (1.01–1.06)**
ECOG performance status (ref: 3–4)				
0–2	1.34 (0.98–1.84)	**3.27 (2.32–4.59)**	0.94 (0.67–1.32)	**2.33 (1.79–3.04)**
Not assessed	**1.97 (1.19–3.27)**	**3.50 (2.04–5.98)**	1.55 (0.89–2.73)	**2.86 (1.77–4.64)**

*Abbreviation*: *ACP* advanced care planning, *ECOG* Eastern Cooperative Oncology Group

^1^Adjusted for both group membership and trajectory covariates. Group membership covariates included age at enrolment, marital status, preferred plan of care, awareness of diagnosis/prognosis by patient/family, medical subsidy means testing, primary decision maker, living arrangement, primary cancer diagnosis, Charlson Comorbidity Index, and ECOG performance status. Trajectory covariates included presence of unstable physical symptoms, presence of distressing psychosocial symptoms, and at least 4 HCA physical visitations per bi-week

^2^Estimates not shown due to extremely small cell count

^3^ACP done, preferred care place and preferred death place were collinear with preferred care plan, and main caregiver was collinear with the primary decision maker. These variables were dropped from the multivariable analysis

*ACP components are (1) preferred place of care, (2) preferred place of death, and (3) preferred plan of care

The following had increased risk of group 3 membership: being younger (RRR 1.05; 95% CI 1.04–1.06 per year decrease), higher CCI (RRR 1.03; 95% CI 1.01–1.06 per score increase), better ECOG (RRR 2.33; 95% CI 1.79–3.04), received 0% medical subsidy (RRR 1.57; 95% CI 1.08–2.28), had haematological malignancy (RRR 4.86; 95% CI 2.20–10.74), had non-blood relations or self as the primary decision maker (RRR 3.78; 95% CI 1.04–13.73), had no ACP (RRR 2.74; 95% CI 1.91–3.93), or had preferred care plans for a full treatment or limited interventions (RRR 2.08; 95% CI 1.31–3.30) (Table 2).

### Association of time-varying needs and visit intensity on healthcare utilization trajectory within groups

Patients assessed with U1 symptoms had a significantly higher incidence of ED visits and hospitalizations in all three groups (ED visits, group 1–3: incidence rate ratio [IRR] 1.74–6.85; hospitalizations, group 1–3: IRR 1.69–6.60). High home visit intensity was significantly associated with a reduced incidence of outpatient visits in all three groups (group 1–3: IRR 0.54–0.84). In addition, high home visit intensity was significantly associated with decreased incidence of ED visits (IRR 0.40; 95% CI 0.25–0.62) and hospitalizations (IRR 0.37; 95% CI 0.24–0.58) in group 2 (Table 3).

**Table 3:** Predictors of acute healthcare utilization trajectories within groups

Variable	Incidence rate ratio (95% CI)								
	ED visitations			Outpatient visitations			Inpatient admissions		
	Group 1	Group 2	Group 3	Group 1	Group 2	Group 3	Group 1	Group 2	Group 3
	Univariable analysis								
Presence of unstable physical symptoms	**7.49 (5.28–10.63)**	**1.40 (1.16–1.69)**	**3.18 (2.57–3.94)**	0.74 (0.54–1.01)	**0.64 (0.48–0.85)**	0.96 (0.86–1.06)	**7.09 (5.08–9.88)**	**1.34 (1.11–1.61)**	**2.39 (2.00–2.86)**
Presence of distressing psychosocial symptoms	**4.12 (1.99–8.53)**	1.14 (0.81–1.61)	**2.15 (1.40–3.31)**	0.92 (0.44–1.90)	0.99 (0.57–1.73)	**0.8 (0.63–1.00)**	**4.66 (2.41–9.00)**	1.16 (0.84–1.61)	**1.81 (1.26–2.60)**
High home visit intensity	**2.70 (1.50–4.84)**	0.77 (0.56–1.07)	**1.76 (1.25–2.49)**	**0.34 (0.19–0.63)**	**0.64 (0.45–0.91)**	**0.82 (0.70–0.96)**	**2.05 (1.10–3.80)**	**0.74 (0.55–1.00)**	**1.44 (1.08–1.92)**
	Multivariable analysis^a^								
Presence of unstable physical symptoms	**6.85 (4.73–9.91)**	**1.74 (1.44–2.1)**	**2.98 (2.38–3.73)**	0.78 (0.56–1.08)	**0.72 (0.54–0.98)**	0.99 (0.89–1.10)	**6.60 (4.59–9.50)**	**1.69 (1.41–2.03)**	**2.35 (1.95–2.83)**
Presence of distressing psychosocial symptoms	1.52 (0.69–3.37)	1.38 (0.92–2.07)	1.50 (1.00–2.25)	1.08 (0.54–2.17)	**1.53 (1.02–2.29)**	**0.78 (0.63–0.97)**	1.89 (0.89–4.01)	**1.52 (1.05–2.22)**	1.37 (0.97–1.94)
High home visit intensity	1.15 (0.68–1.93)	**0.40 (0.25–0.62)**	0.87 (0.61–1.24)	**0.54 (0.29–0.99)**	**0.56 (0.32–0.97)**	**0.84 (0.72–0.99)**	0.89 (0.51–1.56)	**0.37 (0.24–0.58)**	0.84 (0.63–1.13)

^a^Adjusted for both group membership and trajectory covariates. Group membership covariates included age at enrolment, marital status, preferred plan of care, awareness of diagnosis/prognosis by patient/family, medical subsidy means testing, primary decision maker, living arrangement, primary cancer diagnosis, Charlson Comorbidity Index, and ECOG performance status. Trajectory covariates included presence of unstable physical symptoms, presence of distressing psychosocial symptoms, and at least 4 HCA physical visitations per bi-week

## Discussion

We found wide heterogeneity in healthcare utilization within a cohort of cancer patients enrolled in HPC. There appears a place for targeted interventions based on individual trajectories instead of a one-size fits all approach. We identified three distinct patient subgroups: group 1 with constantly low intensity of healthcare utilization; group 2 with persistently high healthcare utilization; and group 3 with early reductions in healthcare utilization that rose gradually over time. Broadly, several predisposing (age, ACP completion, and care preferences), enabling (no medical subsidy, primary decision maker), and need factors (cancer type, comorbidity burden and performance status) were significantly associated with group membership. Severe symptoms resulted in higher incidence rates of ED visits and hospitalizations across all three groups. Higher home visit intensity was associated with reduced outpatient visits across all groups, and reduced rates of ED visits and hospitalizations for group 2. Finer points pertaining to individual groups and their practice and policy implications are discussed next.

Within our cohort, only 53.6% of patients completed ACP, echoing findings from the Kaiser Permanente HomePal program in the USA where only 55% of patients had advance directive documentation. Like HCA, the HomePal program provides interdisciplinary HPC to patients with an estimated prognosis of 12 months or less and allows receipt of concurrent disease-directed therapy [50]. ACP is a process that supports patients in understanding their medical conditions and sharing their personal values, life goals and healthcare preferences with family and healthcare providers [51]. Without documented clarity on patient’s care preferences, it may be difficult for the HPC team to intervene and reduce unnecessary hospital visits [52, 53]. We postulate this as an underlying reason for patients without completed ACP having a higher risk of membership in group 2 or 3. Barriers to ACP may include patient factors (cognitive and emotional barriers), clinician factors (lack of training and prognostication challenges), and system factors (cumbersome documentation process) [54, 55]. More needs to be done to increase ACP completion within HPC services.

Patients with ACP preferences for “active” or “limited” interventions were also at higher risk of being in group 2 or 3 compared with those with ACP preferences for “comfort care only”. However, Singapore’s HPC services do not routinely provide medical interventions such as blood investigations, parenteral hydration or intravenous drug administration [56]. This is unlike some HPC services elsewhere which provide home-based clinical interventions [57, 58]. It is thus unsurprising that documented preferences for medical interventions predispose hospital visits when the need arises. If we desire to provide appropriate goal-concordant care without increasing the burden on hospital resources, a system change may be required. For example, the capacity to provide episodes of hospital-level care within the home could be explored for the treatment of reversible conditions [59]. Indeed, systematic reviews suggest that episodic Hospital-at-Home models were associated with lower costs per care episode without detriment to readmission and mortality rates for various disease groups [60, 61]. Data is however lacking for terminal cancer patients, highlighting areas for future research.

To reduce unplanned ED visits and hospitalizations, another approach would be to pre-emptively intervene for high-risk patients [62]. This may become increasingly possible as risk prediction models improve, following the rise of machine learning for big data analytics [25]. In this study, we found the presence of severe symptoms over 2 weeks to be a significant predictor for increased incidence of ED visits and hospitalizations across all three groups. Time-series symptom data could be explored using predictive modelling for risk-stratified early interventions to manage healthcare utilization.

Lastly, group 2 patients experienced frequent transitions between hospital and home despite having the shortest contact time with HPC before death. For this group, we observed encouraging signals that frequent home visits (≥4 visits per 2 weeks) lowered rates of all healthcare utilization. However, this association was not found for groups 1 and 3 with respect to ED visits and hospitalizations. A previous study demonstrated higher intensity of PC associated with reduced ED visits and hospitalizations in the last 30 days of life [63]. We expanded those early findings by showing the differential impact of HPC on healthcare utilization in three patient groups. Funding model per patient for HPC here is based on monthly block or package rates, similar to non-activity-based funding models in other countries [56, 64]. With the potential of HPC intensity to reduce hospital admissions in persistently high utilizers, considerations could be given to calibrate community funding models based on patient complexity and expected impact on hospital cost reduction.

### Limitations

This study has several limitations. As a retrospective cohort study using readily available EHR data, inherent selection and information biases can occur, implicating the internal validity of results. Our study was conducted within Singapore, which may limit generalizability to HPC elsewhere. However, similarities do exist between Singapore’s HPC model and models of other developed countries, with multidisciplinary home visits, symptom assessment, psychosocial care, and round-the-clock support being common elements [13, 32]. Our sample only included patients referred by SingHealth to HCA, with an underlying assumption that patients previously cared for by SingHealth would continue to seek future care with the same provider. This may limit the generalizability of our findings. We did not capture drug prescription data and primary care utilization, which are additional components of healthcare utilization. Despite this, we believe that the omission of these data did not significantly skew study findings as we have included ED visits, hospitalizations, and outpatient visits that constitute three major components of healthcare costs [3, 4]. Our findings on healthcare utilization trajectories may not extrapolate to non-cancer patients, as compared to cancer counterparts, they may have higher rates of ED use but lower rates of hospitalisations and outpatient visits [65]. While aiming to study trajectories of healthcare utilization from HPC enrolment to death, we did not include patients discharged from HPC. Reasons for discharge included refusal of service, outliving prognosis, and discharge to inpatient hospice. Findings should be interpreted in that light. The optimum GBMTM model was derived based on bi-weekly time-unit intervals, resulting in the exclusion of patients who died within 14 days of HCA enrolment. Thus, our findings are not representative of imminently dying patients referred to HPC. Lastly, further work to externally validate our findings is required.

## Conclusions

This study demonstrated the heterogeneity of healthcare utilization trajectories in HPC patients and identified factors associated with individual trajectories. Our findings suggest the applicability of targeted interventions such as increasing completion of ACP, supporting preferences for medical treatments at home, and proactive symptom interventions. Additionally, home visit intensity could be stratified to accommodate the needs of persistently high healthcare utilizers.

## Supplementary Information

**Additional file 1: Table 1.** Candidate list of variables based on the Andersen’s framework for health service use. **Table 2.** Preferred plan of care categories and definitions**. Table 3.** Comparison of size, income ceiling for eligibility to purchase average price after subsidy of public housing and derived categories of Housing value Index (HI). **Table 4a.** Patient symptom categories**. Table 4b.** Patient psychosocial categories and definitions**. Table 5.** Performance of the optimum model for the trajectories of the composite healthcare utilization measure. **Figure 1.** Trajectories of emergency department (ED) visits using group-based trajectory modelling. Best-fit model based on (A) monthly time-unit, (B) thrice-weekly time-unit, (C) bi-weekly time-unit and (D) weekly time-unit, and (E) the optimum model is the model with bi-weekly time-unit based on trade-off comparison between cohort size and Bayesian Information Criterion (BIC). **Figure 2.** Trajectories of outpatient visits using group-based trajectory modelling. Best-fit model based on (A) monthly time-unit, (B) thrice-weekly time-unit, (C) bi-weekly time-unit and (D) weekly time-unit, and (E) the optimum model is the model with bi-weekly time-unit based on trade-off comparison between cohort size and Bayesian Information Criterion (BIC). **Figure 3.** Trajectories of hospitalizations using group-based trajectory modelling. Best-fit model based on (A) monthly time-unit, (B) thrice-weekly time-unit, (C) bi-weekly time-unit and (D) weekly time-unit, and (E) the optimum model is the model with bi-weekly time-unit based on trade-off comparison between cohort size and Bayesian Information Criterion (BIC). Supplementary Information on Group-based trajectory modelling (GBTM) and Group-based multi-trajectory modelling (GBMTM) Analyses.

## Data Availability

The anonymized datasets used and/or analysed during the current study are available from the corresponding author on reasonable request

## Abbreviations

EOL: End-of-life
HPC: Home-based palliative care
HCA: HCA Hospice
ACP: Advance care plan
SingHealth RHS: Singapore Health Services Regional Health System
ED: Emergency department
GBTM: Group-based trajectory modelling
GBMTM: Group-based multi-trajectory modelling
