# Supplementary material for: Longitudinal patterns and predictors of healthcare utilization among cancer patients on home-based palliative care in Singapore: a group-based multi-trajectory analysis
Source: BMC Med. 2022 Sep 22;20:313. doi: 10.1186/s12916-022-02513-y (PMC9494890; doi:10.1186/s12916-022-02513-y)
Supplement: Supplementary file 1 — Additional file 1: Table 1. Candidate list of variables based on the Andersen’s framework for health service use. Table 2. Preferred plan of care categories and definitions. Table 3. Comparison of size, income ceiling for eligibility to purchase average price after subsidy of public housing and derived categories of Housing value Index (HI). Table 4a. Patient symptom categories. Table 4b. Patient psychosocial categories and definitions. Table 5. Performance of the optimum model for the trajectories of the composite healthcare utilization measure. Figure 1. Trajectories of emergency department (ED) visits using group-based trajectory modelling. Best-fit model based on (A) monthly time-unit, (B) thrice-weekly time-unit, (C) bi-weekly time-unit and (D) weekly time-unit, and (E) the optimum model is the model with bi-weekly time-unit based on trade-off comparison between cohort size and Bayesian Information Criterion (BIC). Figure 2. Trajectories of outpatient visits using group-based trajectory modelling. Best-fit model based on (A) monthly time-unit, (B) thrice-weekly time-unit, (C) bi-weekly time-unit and (D) weekly time-unit, and (E) the optimum model is the model with bi-weekly time-unit based on trade-off comparison between cohort size and Bayesian Information Criterion (BIC). Figure 3. Trajectories of hospitalizations using group-based trajectory modelling. Best-fit model based on (A) monthly time-unit, (B) thrice-weekly time-unit, (C) bi-weekly time-unit and (D) weekly time-unit, and (E) the optimum model is the model with bi-weekly time-unit based on trade-off comparison between cohort size and Bayesian Information Criterion (BIC). Supplementary Information on Group-based trajectory modelling (GBTM) and Group-based multi-trajectory modelling (GBMTM) Analyses. [file 12916_2022_2513_MOESM1_ESM.docx]

**Additional File 1**

Table 1: Candidate list of variables based on the Andersen’s framework for health service use

| **Factor Group** | **Variable Group** | **Variable** |
| --- | --- | --- |
| Predisposing characteristics | Sociodemographic | Age at enrolment, years |
|  |  | Gender |
|  |  | Male |
|  |  | Female |
|  |  | Ethnicity |
|  |  | Chinese |
|  |  | Malay |
|  |  | Indian |
|  |  | Others |
|  |  | Religion |
|  |  | Christianity (all denominations) |
|  |  | Buddhism |
|  |  | Taoism |
|  |  | Islam |
|  |  | Others |
|  |  | No religion |
|  |  | Missing |
|  |  | Marital status |
|  |  | Single |
|  |  | Married |
|  |  | Separated / Divorced |
|  |  | Widowed |
|  |  | Residential status |
|  |  | Singapore citizen |
|  |  | Permanent resident |
|  | Health Beliefs | At least one component of ACP^1^ done at baseline |
|  |  | Yes |
|  |  | Preferred place of care at baseline |
|  |  | Hospital |
|  |  | Home |
|  |  | Others |
|  |  | Missing |
|  |  | ACP not done |
|  |  | Preferred place of death at baseline |
|  |  | Home |
|  |  | Hospice |
|  |  | Others |
|  |  | Missing |
|  |  | ACP not done |
|  |  | Preferred plan of care at baseline |
|  |  | Full active |
|  |  | Limited intervention |
|  |  | Comfort only |
|  |  | Missing |
|  |  | ACP not done |
|  |  | Patient awareness of diagnosis at baseline |
|  |  | No |
|  |  | Yes |
|  |  | Missing |
|  |  | Patient awareness of prognosis at baseline |
|  |  | No |
|  |  | Yes |
|  |  | Missing |
|  |  | Family awareness of diagnosis at baseline |
|  |  | No |
|  |  | Yes |
|  |  | Missing |
|  |  | Family awareness of prognosis at baseline |
|  |  | No |
|  |  | Yes |
|  |  | Missing |
| Enabling resources | Socioeconomic status | Housing value Index (HI) |
|  |  | HI-low (high subsidy) |
|  |  | HI-med (moderate subsidy) |
|  |  | HI-high (minimal or no subsidy) |
|  |  | Others (nursing home, sheltered homes, etc) |
|  |  | Medical subsidy means testing |
|  |  | 0% (no subsidy) |
|  |  | 1% - 25% |
|  |  | 26% – 50% |
|  |  | 51% – 80% |
|  |  | Not done |
|  | Social Structure | Main caregiver |
|  |  | Self |
|  |  | Spouse |
|  |  | Relatives (including children) |
|  |  | Others |
|  |  | Missing |
|  |  | Primary decision maker |
|  |  | Self |
|  |  | Spouse |
|  |  | Children |
|  |  | Relatives |
|  |  | Others |
|  |  | Missing |
|  |  | Living arrangement |
|  |  | Alone |
|  |  | With spouse only |
|  |  | With children only |
|  |  | With spouse and children |
|  |  | With relatives |
|  |  | Others |
|  |  | Missing |
| Need factors for care | Symptom and Psychosocial Needs  (Time-varying variable) | Presence of U1 symptoms within 2 weeks^2^ |
|  |  | Yes |
|  |  | No |
|  |  | Presence of P3 or P4 symptoms within 2 weeks^3^ |
|  |  | Yes |
|  |  | No |
|  | Clinical Characteristics | Primary cancer diagnosis |
|  |  | Lip, oral cavity and pharynx |
|  |  | Digestive organs |
|  |  | Respiratory and intrathoracic |
|  |  | Breast |
|  |  | Female genital organs |
|  |  | Male genital organs |
|  |  | Urinary tract |
|  |  | Lymphoid, hematopoietic, and related tissue |
|  |  | Others |
|  |  | Charlson Comorbidity Index (CCI) at enrolment |
|  |  | ECOG^4^ at enrolment |
|  |  | 0 – 2 |
|  |  | 3 – 4 |
|  |  | Not done |
|  |  | Missing |

^1^ACP = Advance Care Plan (components are 1) Preferred place of care, 2) Preferred place of death, and 3) Preferred plan of care); ^2^U1 = Clinically unwell with acute unstable symptoms such as acute pain; ^3^P3 = Signs of inability to cope with rising psychological and spiritual distress; ^3^P4 = Acute psychological distress impacting patient care with possible indicators of neglect, abuse, or suicidal ideation; ^4^ECOG = Eastern Cooperative Oncology Group

Table 2: Preferred plan of care categories and definitions

| **Comfort measures only** | Patient is to be treated with dignity and respect. Reasonable measures are made to offer food and fluids. Medications, oxygen, and other measures may be used as needed for comfort. Do not intubate. These measures may be used where the patient resides. Consider transfer only if comfort needs cannot be met in current location. |
| --- | --- |
| **Limited interventions** | Include care described above. To initiate limited trial of treatment. May include oral/intravenous medications. Continue with comfort measures if there is no clinical improvement. Do not use endotracheal intubation or long-term life support measures. May consider non-invasive ventilation support. Transfer to hospital if indicated. Avoid transfer to intensive care unit. |
| **Full treatment** | Includes care described above. May consider intubation, mechanical ventilation, and cardioversion. Management may include transfer to intensive care if indicated. These measures are subject to the assessment and decisions of the hospital care. |

Table 3: Comparison of size, income ceiling for eligibility to purchase average price after subsidy of public housing and derived categories of Housing value Index (HI).

| **Apartment Type** | **Average Size (m^2^)** | **Income Ceiling (SGD/Month)** | **Average price after subsidy (SGD)** | **Housing value Index (HI) category** |
| --- | --- | --- | --- | --- |
| 1 – 2 rooms | 33-45 | 1 500 | 43 000 | HI (low) |
| 3 rooms | 65 | 4 000 – 8 000 | 132 000 | HI (med) |
| 4 rooms | 90 | 12 000 – 18 000 | 270 000 |  |
| 5 rooms | 110 | 12 000 – 18 000 | 396 000 | HI (high) |
| Executive | 130 | 12 000 – 18 000 | >396 000 |  |
| Private Housing | ~85 | Nil | 1 250 000 |  |

Replicated with permission from Wong FY, Wong RX, Zhou S, Ong WS, Pek PP, Yap Y-S, et al. Effects of housing value and medical subsidy on treatment and outcomes of breast cancer patients in Singapore: A retrospective cohort study. Lancet Reg Health West Pac. 2021 Jan;6:100065.

Table 4a: Patient symptom categories

| **Category** | **Description** |
| --- | --- |
| **S0**  Stable-stable | Asymptomatic with no other problems or outstanding issues |
| **S1**  (Stable – chair/bedbound)  Stable – appliance  Stable – poor social support | Dependent for activities of daily living (e.g. hemiplegia, paraplegia, quadriplegia)  Stable but with an appliance such as urinary catheter, stomas, nasogastric tube, drainage tubes, dressings for chronic wounds.  Living alone or all family working during the day |
| **S2**  Stable-active | Active disease. Symptoms controlled. Recently in unstable category. |
| **U1**  Unstable | Acute symptoms such as uncontrolled pain, anorexia, dehydration, nausea, vomiting, acute urinary retention and constipation  New onset confusion or delirium  High fever, especially on concurrent chemotherapy.  Uncontrolled dyspnea or new onset of dyspnea.  Acute onset of weakness  Recent fall  Any emotional, social crisis such as suicidal intent |
| **U2**  Deteriorating / Terminal | On Care of Dying pathway  High chance of dying in days or within a week |

Table 4b: Patient psychosocial categories and definitions

| **Category** | **Description** |
| --- | --- |
| **P1**  Stable | - Able to function and cope well in most biopsychosocial and spiritual (BPSS) aspects - Good support system - In control of BPSS and life’s issues |
| **P2**  Observe | - Able to function and cope well generally - Some indicators of BPSS concerns that requires monitoring such as low mood, care concerns, financial concerns. |
| **P3**  Active | - Signs of inability to cope - Distress arising from symptoms due to illness progression or health condition - Signs of mental health, psychological and spiritual distress - Presence of social stressors that affects coping - Presence of ethical concerns and dilemmas that affect patient’s quality of life and care - Presence of indicators that can affect bereavement |
| **P4**  Very active | - Acute psychosocial and spiritual distress arising from pain, symptoms, and psychosocial stressors - Relationship conflict/stressors impacting immediate care and caregiver coping - Indicators of neglect and abuse - Suicide ideation |

Table 5. Performance of the optimum model for the trajectories of the composite healthcare utilization measure

| Trajectory group | Estimated group size by model, % | No. of patients classified into group | % of patients classified into group | Average posterior probabilities of group membership |
| --- | --- | --- | --- | --- |
| 1 | 31.9 | 551 | 35.1 | 0.83 |
| 2 | 44.1 | 664 | 42.2 | 0.88 |
| 3 | 24.0 | 357 | 22.7 | 0.90 |
| Total | 100.0 | 1572 | 100.0 |  |

Figure 1: Trajectories of emergency department (ED) visits using group-based trajectory modelling. Best-fit model based on (A) monthly time-unit, (B) thrice-weekly time-unit, (C) bi-weekly time-unit and (D) weekly time-unit, and (E) the optimum model is the model with bi-weekly time-unit based on trade-off comparison between cohort size and Bayesian Information Criterion (BIC).


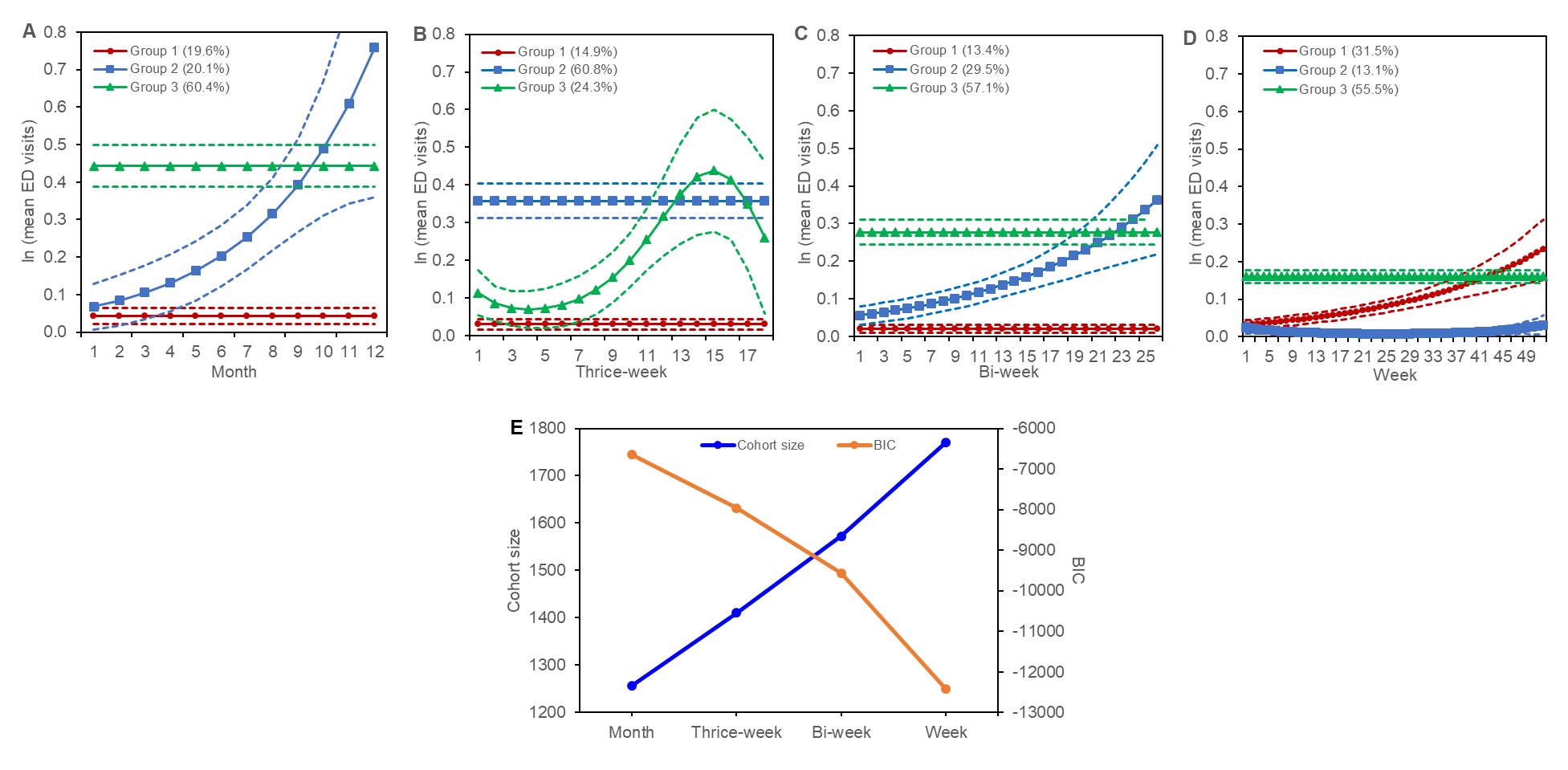


Figure 2: Trajectories of outpatient visits using group-based trajectory modelling. Best-fit model based on (A) monthly time-unit, (B) thrice-weekly time-unit, (C) bi-weekly time-unit and (D) weekly time-unit, and (E) the optimum model is the model with bi-weekly time-unit based on trade-off comparison between cohort size and Bayesian Information Criterion (BIC).


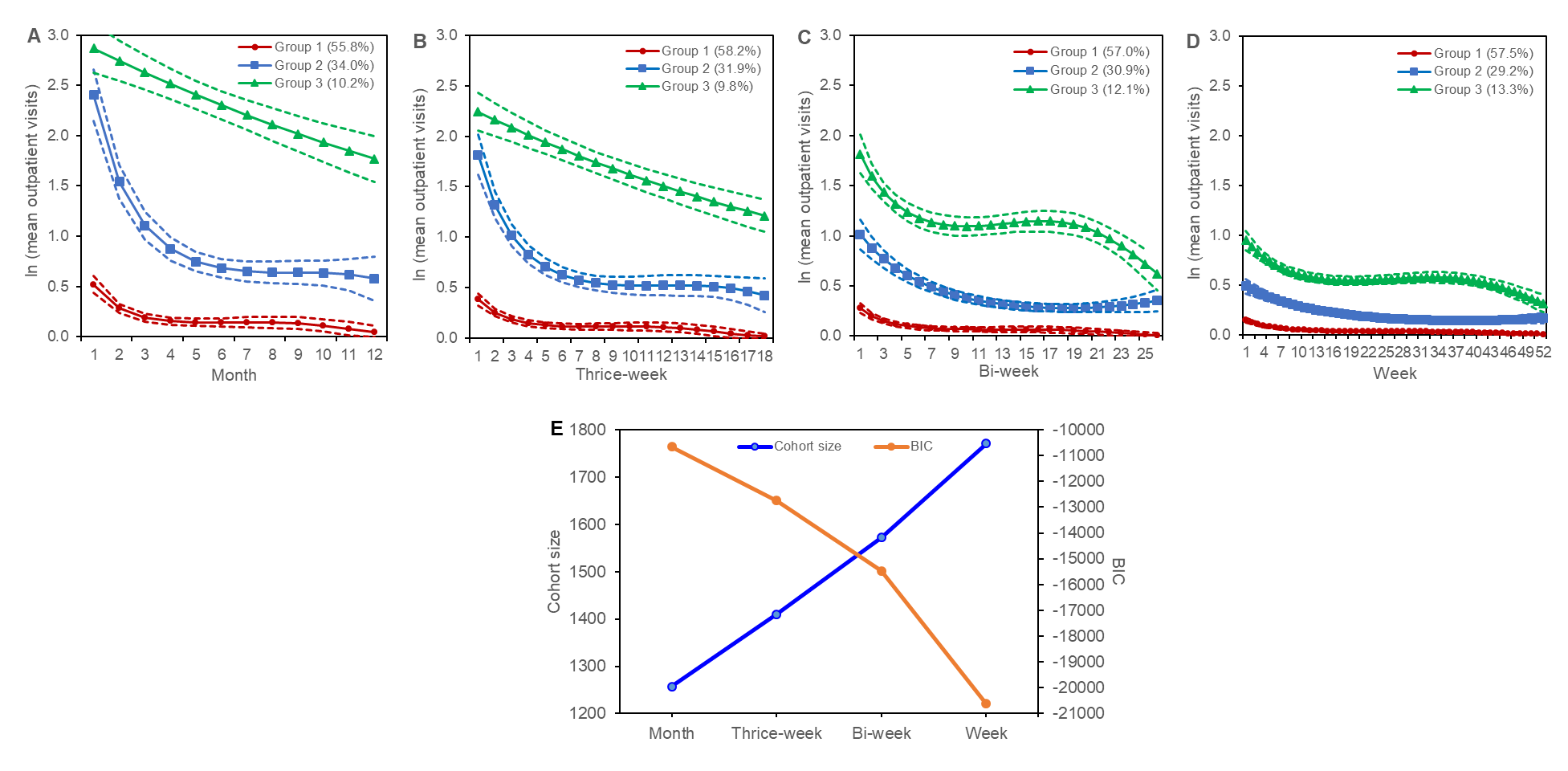


Figure 3: Trajectories of hospitalizations using group-based trajectory modelling. Best-fit model based on (A) monthly time-unit, (B) thrice-weekly time-unit, (C) bi-weekly time-unit and (D) weekly time-unit, and (E) the optimum model is the model with bi-weekly time-unit based on trade-off comparison between cohort size and Bayesian Information Criterion (BIC).


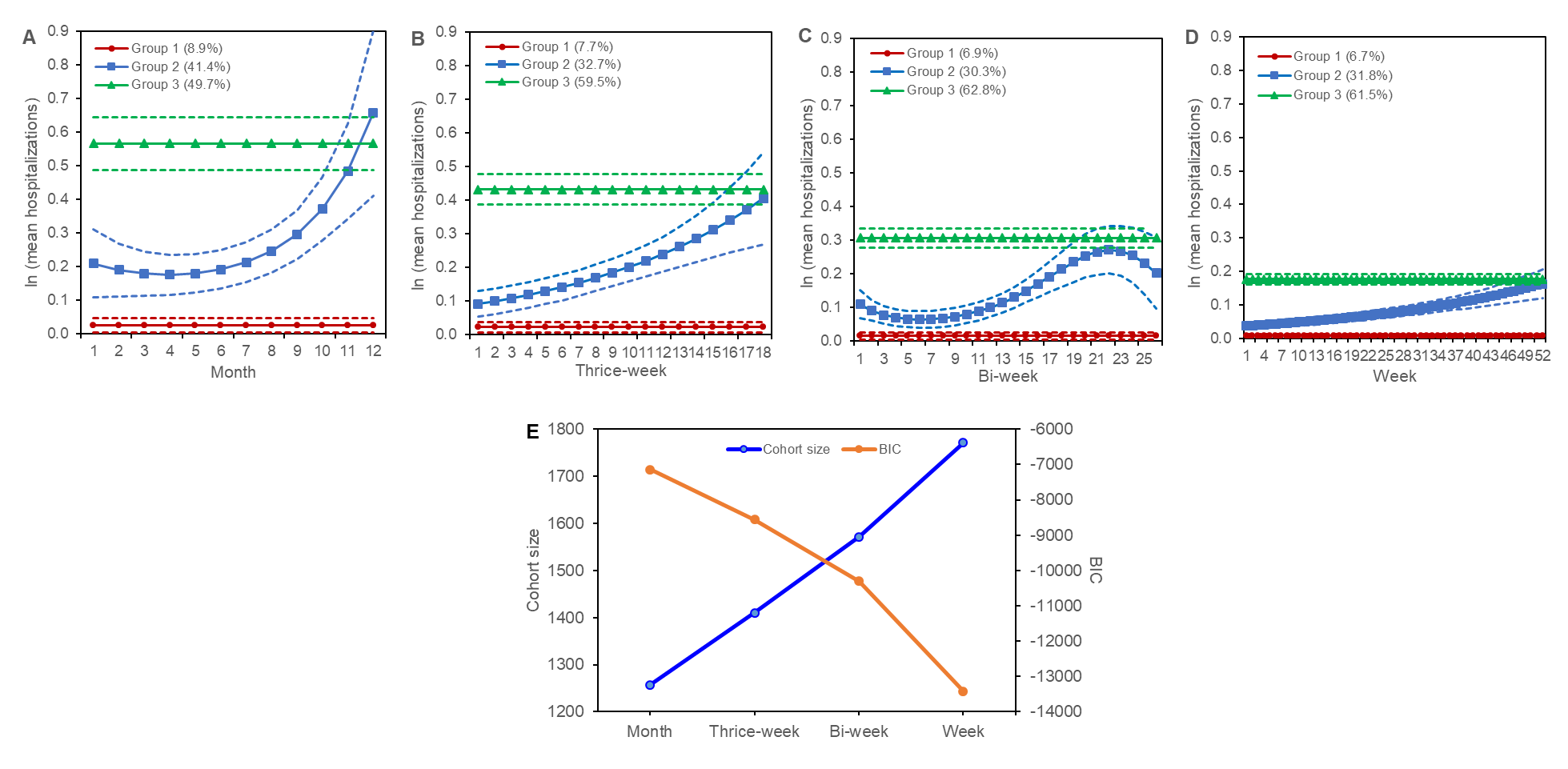


Supplementary Information on Group-based trajectory modelling (GBTM) and Group-based multi-trajectory modelling (GBMTM) Analyses

GBTM is an application of finite mixture modelling in which maximum likelihood estimation is used to identify latent subgroups of individuals who follow similar trajectories for a variable over time.^1^ We applied GBTM to determine trajectories for the number of emergency department (ED) visits, hospitalizations and outpatient visits individually, with extensions to account for non-random patient attrition due to death.^2^ This means that the trajectories of each of these measures is modelled jointly with the probability of dying, and the estimated probability of each trajectory group by the model is the estimated size of the group at baseline.

We used Poisson distribution to model each measure with time as an independent variable. We examined four time-units viz. weekly, bi-weekly, thrice-weekly and monthly, as we hypothesized that relevant changes in health service usage over the 1-year study period would occur over weeks rather than days. For each time-unit analysis, we excluded patients who passed away within the first time-unit interval. The shape of each trajectory group in GBTM is specified by a polynomial function of time-unit. To determine the optimum number of groups and trajectory shape of each group, we fitted all possible models for 2-3 groups with the order of polynomial for the time variable varied between 0 and 3. We kept the number of groups to 3, as models fitted with 4 groups showed extensive overlapping between some of the fitted trajectory groups and there were frequently less than 5% of patients estimated for the smallest group. We kept the maximum polynomial order to 3 as this order has been suggested to be sufficiently flexible to capture meaningful changes when modelling about 12-15 months of data.^3^ Among fitted models with statistical significant trajectory shape for all the groups in the model, we selected the one with the highest Bayesian Information Criteria (BIC) as the best fit model for the time-unit. To determine the optimum model for each healthcare utilization measure, we compared the cohort size and BIC between the best fit model of the 4 time-units and identified the time-unit that provided a balance between information loss and goodness of model fit.

GBMTM is a generalization of GBTM in that it identifies latent clusters of individuals following similar trajectories across multiple variables of an outcome of interest.^4^ We applied GBMTM to identify distinct trajectories for the composite outcome of the number of ED, hospitalizations, and outpatient visits. Based on the narrowest time-unit interval and the highest number of groups across the optimum GBTM model of the 3 measures, we fit all possible models with a maximum polynomial order of 3 using GBMTM. Following a similar approach as the GBTM analyses, we selected the model with the highest BIC as the optimum GBMTM model for the composite outcome measure. We classified patients into one of the trajectory groups in the optimum GBMTM model based on the maximum posterior probability assignment rule. We assessed the performance of the optimum GBMTM model using these criteria: 1) close correspondence between model’s estimated group size and actual percentage of patients classified into each group, 2) high (>0.7) average posterior probabilities of group membership, 3) enough patients (>5% in proportion) classified in each group, and 4) reasonably narrow confidence band for each group.

1. Nagin DS. *Group-Based Modeling of Development*. Harvard University Press; 2005.

2. Haviland AM, Jones BL, Nagin DS. Group-based Trajectory Modeling Extended to Account for Nonrandom Participant Attrition. *Sociological Methods & Research*. 2011;40(2):367-390. doi:10.1177/0049124111400041

3. Franklin JM, Shrank WH, Pakes J, et al. Group-based trajectory models: a new approach to classifying and predicting long-term medication adherence. *Med Care*. 2013;51(9):789-796. doi:10.1097/MLR.0b013e3182984c1f

4. Nagin DS, Jones BL, Passos VL, Tremblay RE. Group-based multi-trajectory modeling. *Stat Methods Med Res*. 2018;27(7):2015-2023. doi:10.1177/0962280216673085
